# The clinical and cost effectiveness of surgical interventions for stones in the lower pole of the kidney: the percutaneous nephrolithotomy, flexible ureterorenoscopy and extracorporeal shockwave lithotripsy for lower pole kidney stones randomised controlled trial (PUrE RCT) protocol

**DOI:** 10.1186/s13063-020-04326-x

**Published:** 2020-06-04

**Authors:** Sam McClinton, Kathryn Starr, Ruth Thomas, Graeme MacLennan, Thomas Lam, Rodolfo Hernandez, Robert Pickard, Ken Anson, Terry Clark, Steven MacLennan, David Thomas, Daron Smith, Ben Turney, Alison McDonald, Sarah Cameron, Oliver Wiseman

**Affiliations:** 1 Aberdeen Royal Infirmary, Foresterhill, Aberdeen, UK; 2 Academic Urology Unit, University of Aberdeen, Health Sciences Building, Foresterhill, Aberdeen, UK; 3 Health Services Research Unit, University of Aberdeen, Health Sciences Building, Foresterhill, Aberdeen, UK; 4 Health Economics Research Unit, University of Aberdeen, Polwarth Building, Foresterhill, Aberdeen, UK; 5 Institute of Cellular Medicine, Newcastle University, Newcastle Upon Tyne, UK; 6 St George’s Hospital, London, UK; 7 Stone Patient Advisory Group, Section of Endourology, British Association of Urological Surgeons, London, UK; 8 Newcastle upon Tyne Hospitals NHS Foundation Trust, Newcastle Upon Tyne, UK; 9 University College London Hospitals NHS Foundation Trust, London, UK; 10 Oxford University Hospitals NHS Trust, Headley Way, Oxford, UK; 11 Addenbrooke’s NHS Trust, Hills Road, Cambridge, UK

**Keywords:** Renal stone, Lower pole stone, Percutaneous nephrolithotomy, Flexible ureterorenoscopy, Extracorporeal shockwave lithotripsy

## Abstract

**Introduction:**

Renal stones are common, with a lifetime prevalence of 10% in adults. Global incidence is increasing due to increases in obesity and diabetes, with these patient populations being more likely to suffer renal stone disease. Flank pain from stones (renal colic) is the most common cause of emergency admission to UK urology departments. Stones most commonly develop in the lower pole of the kidney (in ~35% of cases) and here are least likely to pass without intervention. Currently there are three technologies available within the UK National Health Service to remove lower pole kidney stones: extracorporeal shockwave lithotripsy (ESWL), percutaneous nephrolithotomy (PCNL) and flexible ureterorenoscopy (FURS) with laser lithotripsy. Current evidence indicates there is uncertainty regarding the management of lower pole stones, and each treatment has advantages and disadvantages. The aim of this trial is to determine the clinical and cost effectiveness of FURS compared with ESWL or PCNL in the treatment of lower pole kidney stones.

**Methods:**

The PUrE (PCNL, FURS and ESWL for lower pole kidney stones) trial is a multi-centre, randomised controlled trial (RCT) evaluating FURS versus ESWL or PCNL for lower pole kidney stones. Patients aged ≥16 years with a stone(s) in the lower pole of either kidney confirmed by non-contrast computed tomography of the kidney, ureter and bladder (CTKUB) and requiring treatment for a stone ≤10 mm will be randomised to receive FURS or ESWL (RCT1), and those requiring treatment for a stone >10 mm to ≤25 mm will be randomised to receive FURS or PCNL (RCT2). Participants will undergo follow-up by questionnaires every week up to 12 weeks post-intervention and at 12 months post-randomisation. The primary clinical outcome is health status measured by the area under the curve calculated from multiple measurements of the EuroQol five dimensions five-level version (EQ-5D-5L) questionnaire up to 12 weeks post-intervention. The primary economic outcome is the incremental cost per quality-adjusted life year gained at 12 months post-randomisation.

**Discussion:**

The PUrE trial aims to provide robust evidence on health status, quality of life, clinical outcomes and resource use to directly inform choice and National Health Service provision of the three treatment options.

**Trial registration:**

ISRCTN: ISRCTN98970319. Registered on 11 November 2015.

## Introduction

### Background

Renal tract stone disease is very common, with a lifetime prevalence of approximately 10% in the adult population across the world [1]. It mainly affects adults of working age and the incidence has been increasing over the past decades [2, 3]. This increase is partly due to people with obesity or diabetes being more likely to suffer renal stone disease and results in a higher burden for health care and associated costs for high-resource countries [4]. Approximately 50% of people with renal tract stones will experience symptoms, typically kidney pain, and about 25% of patients with stones will require active treatment [5–7]. Some people with stones can develop more serious problems including uncontrolled pain, infection, visible blood in the urine (haematuria), impaired kidney function and kidney failure. Despite successful removal of the initial stone, any treated patients may develop a further stone, with a lifetime recurrence risk of 50% [8]. Renal stones are a major burden on the National Health Service (NHS) in the UK resulting in over 82,000 inpatient hospital stays and over 25,000 procedures carried out to remove stones in England in 2013–2014 [9]. Kidney pain from stones (renal colic) is the most common cause of emergency admission to urology departments in the UK and, given the age group most commonly affected, results in time off work and loss of economic activity [3]. The ongoing need for pain killers and the detriment to family, social and work activity reduces quality of life and incurs additional costs.

Stones most commonly develop in the lower part (pole) of the kidney, accounting for up to 35% of cases [10]. There are currently three technologies available within the NHS to remove lower pole kidney stones: extracorporeal shockwave lithotripsy (ESWL), percutaneous nephrolithotomy (PCNL) and flexible ureterorenoscopy (FURS) with laser lithotripsy. The choice of treatment can be guided by stone size, likely stone composition, the anatomy of the drainage system of the affected kidney, clinician and patient preference, and availability of equipment and expertise [11]. Current evidence indicates that the success rate in terms of stone clearance differs between these technologies, which may partly be related to stone size. They are also distinct in terms of the degree of invasiveness, anaesthetic requirement, treatment setting, number of procedures required to clear the stone, and type and rate of complications [11, 12].

ESWL is non-invasive, has a low risk of complications, and does not require anaesthesia. Current evidence suggests it has reasonable efficacy in terms of stone clearance for smaller lower pole stones at 3 months (63–74% clearance rate for stones ≤10 mm) [10]. However, 3-month efficacy rates for lower pole stones >10 mm appear to be lower (23–56% for 11–20 mm stones, and 14–33% for 21–30 mm stones) [13, 14]. If the stone is not cleared then additional treatments may be required using either repeated ESWL or more invasive options. Following ESWL, small residual stone fragments can be left in the kidney and may result in recurrent stone formation over time (20% at 5 years) [7, 15].

Having considered this evidence, guidance issued by the European Association of Urology and widely followed in UK clinical practice recommends ESWL as an option for lower pole stones ≤10 mm, whereas for larger stones the recommended options are FURS or PCNL [11]. However the guidance adds that ESWL may be used for larger stones if stone factors and patient preference are favourable. Flexible ureteroscopy and laser fragmentation and PCNL are more invasive than ESWL, require a general anaesthetic, and carry a greater risk of complications [16, 17]. A single FURS treatment appears to result in a good clearance rate for stones up to 15 mm, with repeat procedures or combined procedures required for larger stones. PCNL is the most invasive treatment option and is associated with a higher risk of complications, but it also appears to result in the highest stone clearance rates which are close to 100% for stones ≤10 mm, 93% for stones 11–20 mm and 86% for stones 21–30 mm [18]. Stone clearance rates for FURS appear to lie between those of ESWL and PCNL [19–25]. The European Association of Urology guidance also comments that there remains considerable uncertainty regarding the management of lower pole stones, with each treatment option having advantages and disadvantages.

### Rationale for the trial

A Cochrane review and meta-analysis in 2014 of randomised controlled trials (RCTs) compared ESWL with either FURS or PCNL for the treatment of renal stones [12]. The review concluded that PCNL had a better stone-free rate than ESWL at 3 months (relative risk (RR) 0.39, 95% confidence interval (CI) 0.27–0.56), whereas FURS appeared to have similar stone-free rates to ESWL (RR 0.91, 95% CI 0.64–1.30). The meta-analysis included five RCTs (*n* = 338); however, only three focused on lower pole stones. Of these three RCTs (160 participants), two compared ESWL with PCNL, one for stones up to 30 mm^13^ and one for stones up to 20 mm [26]. The third compared ESWL with FURS for lower pole stones ≤10 mm [27]. The review concluded that the included trials were small and of low methodological quality. The authors had planned to undertake subgroup analyses by size and location of stone, but this was not done “because of insufficient data”.

**Fig. 1:**
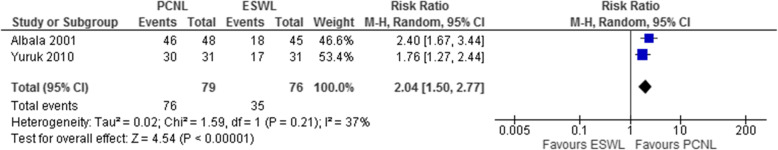
Forest plot demonstrating the meta-analysis of percutaneous nephrolithotomy (PCNL) versus extracorporeal shockwave lithotripsy (ESWL) for the outcome of stone-free rate at 3 months for lower pole stones ≤20mm. Albala and colleagues (2001) [13] and Yuruk and colleagues (2010) [26] reported outcomes for lower pole stones <20 mm. Albala and colleagues [13] also reported outcomes for stones ≤10 mm and 11–20 mm (see Table 1 in Donaldson and colleagues [28]). CI confidence interval, df degrees of freedom, M-H Mantel–Haenszel

**Fig. 2:**
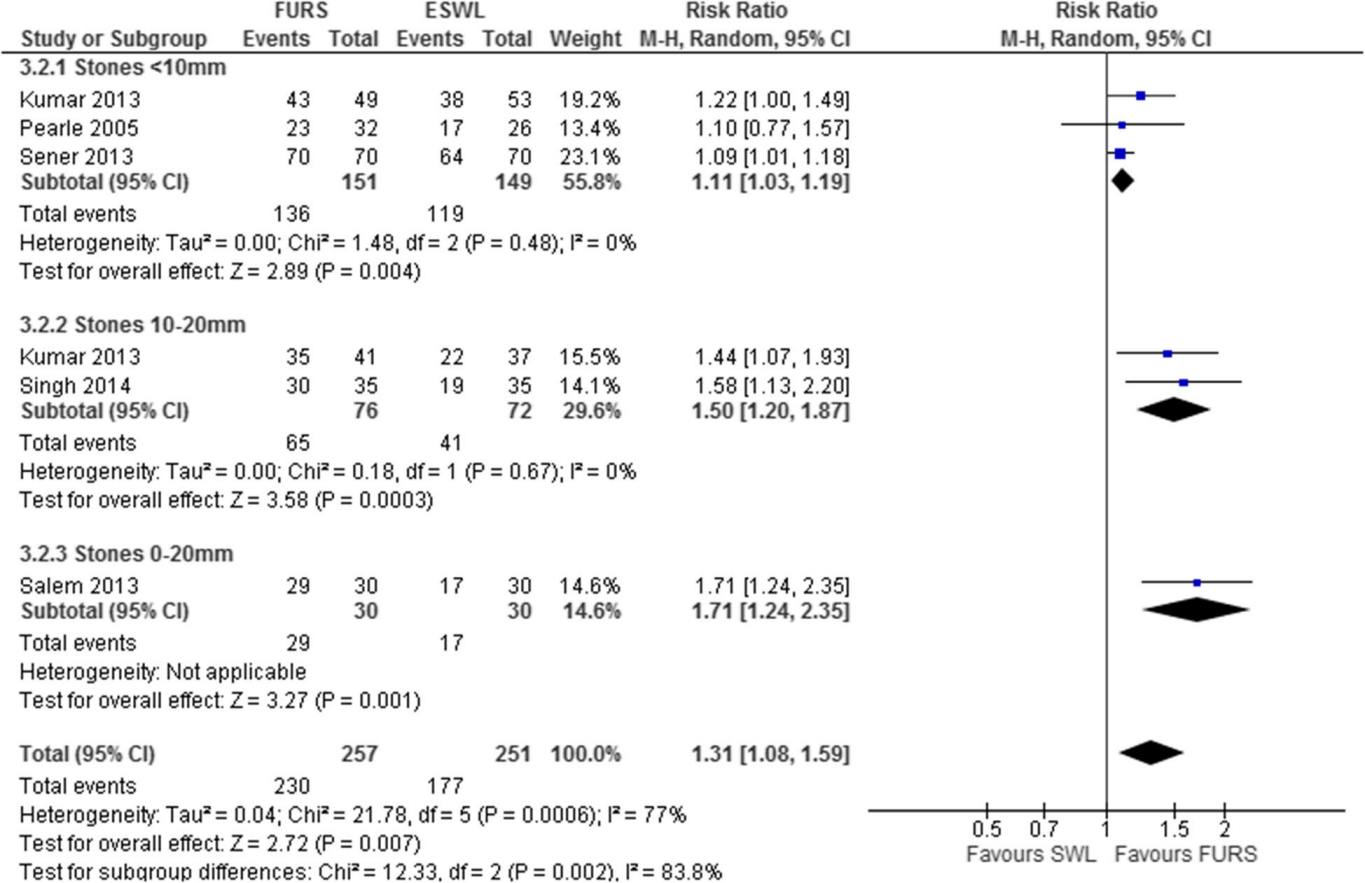
Forest plot demonstrating meta-analysis of flexible ureterorenoscopy (FURS) versus extracorporeal shockwave lithotripsy (ESWL) for the outcome of stone-free rate for lower pole stones at 3 months. Sener and colleagues (2014) [32] and Pearle and colleagues (2005) [27] included stones ≤10mm; Singh and colleagues (2014) [33] included stones 10–20 mm; Kumar and colleagues (2013) [29] and Salem and colleagues (2013) [31] included stones ≤20 mm. Kumar and colleagues [29] reported results for stones 0–9.99 mm and 10–20 mm individually, whilst Salem and colleagues [31] only reported results for stones ≤20 mm. All studies reported the stone-free rate at 3 months, except Singh and colleagues [33] who reported the stone-free rate at 1 month. CI confidence interval, df degrees of freedom, M-H Mantel–Haenszel

A systematic review performed by some of the PUrE (PCNL, FURS and ESWL for lower pole kidney stones) investigators [28] focused solely on stones located in the lower pole of the kidney, and included trials comparing PCNL with FURS (a comparison not considered in the Cochrane review). This review identified four additional relevant trials involving 408 participants [29–32] and we undertook subgroup analyses by stone size (<10 mm and 10–20 mm). Looking at the seven trials involving participants with lower pole kidney stones as a whole, the Grading of Recommendations Assessment, Development and Evaluation certainty of evidence ratings for the outcome of stone-free rates indicated they were of ‘moderate’ quality. Our meta-analyses found PCNL and FURS produced significantly higher stone-free rates than ESWL for lower pole stones ≤20 mm at 3 months. Combining two RCTs (*n* = 155), stone-free rates for those participants with stones ≤20 mm were higher following PCNL than ESWL (RR 2.04, 95% CI 1.50–2.77; Fig. 1). Combining five RCTs (*n* = 508) showed that FURS resulted in a higher stone clearance rate than ESWL (RR 1.31, 95% CI 1.08–1.59; Fig. 2). However, in a subgroup meta-analysis combining three studies (*n* = 300) for stones ≤10 mm the advantage of FURS over ESWL was less, although still statistically significant (RR 1.11, 95%CI 1.03–1.19; Fig. 2). One RCT (*n* = 93) which reported the stone-free rate categorised by stone size for PCNL versus ESWL found that the degree of superiority of PCNL was lower for stones ≤10 mm compared to those >10 mm to ≤20 mm (RR 1.56, 95% CI 1.11–2.21 versus RR 2.40, 95% CI 1.67–3.44; Fig. 2). Although stone-free rates were higher when treated with PCNL than with FURS, there was considerable uncertainty around this estimate as the data came from only one small RCT (*n* = 28) [30].

The included trials reported few data on patient outcomes (such as quality of life) or on resource use, and none on cost effectiveness. Pearle and colleagues [27] suggested that ESWL gave better quality of life, shorter convalescence (days to 100% recovered), and had fewer analgesic requirements than FURS (participants had lower pole stones ≤10 mm). Conversely, Singh and colleagues [33] reported significantly higher participant satisfaction with FURS and comparable convalescence (time to return to routine activity) after having three or fewer ESWL sessions (participants had stone sizes of 10–20 mm). Convalescence was shorter after just a single ESWL session. There were conflicting data on patients’ willingness to undergo the procedure again. In one trial [27] the participants favoured ESWL, whereas in another [33] FURS was preferred. ESWL (one session) was associated with a shorter hospital stay than either PCNL [13] or FURS [27]. One trial also suggested shorter treatment duration for ESWL (one session) than FURS [27].

In summary, there is some evidence to inform estimates of the relative clinical effectiveness (based on the stone-free rate) of ESWL, FURS and PCNL in the treatment of lower pole stones and to guide clinical practice, but for most outcomes there is only moderate or low certainty in the evidence [28]. There is sparse evidence on the impact of these treatments on patient-reported health status and quality of life outcomes (such as severity and duration of pain after intervention), their care pathway (such as the need for additional treatments) and resource use. The PUrE trial aims to provide robust evidence on health status, quality of life, clinical outcomes and resource use to both the NHS and society to close this gap in evidence. The results will benefit patients, clinicians and the NHS as it will inform provision, guidance and decision-making in regard to which of the competing interventions (ESWL, FURS or PCNL) is the most suitable (clinically effective and cost effective) for the treatment of people with lower pole kidney stones of varying sizes.

## Methods

### Trial objectives

The aim of this study is to determine which of FURS, PCNL and ESWL offers the best treatment outcomes in terms of clinical effectiveness and cost effectiveness for people with lower pole kidney stones seeking treatment within the UK NHS. An initial pilot phase will be built in to the trial to assess feasibility of recruitment and check appropriateness of eligibility criteria and outcome measures. The research question to be addressed is: in people requiring treatment for lower pole stones of the kidney does FURS with laser lithotripsy result in better quality of life than standard treatment with ESWL or PCNL according to stone size, and is it cost-effective for the UK NHS?

The clinical effectiveness and cost effectiveness of FURS as the first treatment option in comparison to ESWL for stones ≤10 mm in maximum dimension or PCNL for stones >10 mm and ≤25 mm in maximum dimension will be determined with respect to: 1) patient-reported health status measured as area under the curve (AUC) of the EuroQol five dimensions five-level version (EQ-5D-5L) questionnaire completed at multiple time points up to 12 weeks post-intervention; 2) incremental cost per quality-adjusted life year (QALY) at 12 months post-randomisation; 3) successful stone clearance at 12 weeks; 4) further interventions required to treat stones within 12 months of randomisation; and 5) treatment-related harms experienced up to 12 months after randomisation.

The null hypotheses being tested are: 1) the use of FURS to treat lower pole kidney stones ≤10 mm will not be different to ESWL as assessed by the EQ-5D-5L AUC up to 12 weeks post-treatment; 2) the use of FURS to treat lower pole stones of the kidney >10 mm and ≤25 mm will not be different to PCNL as assessed by the EQ-5D-5L AUC up to 12 weeks post-treatment.

### Trial design

**Fig. 3:**
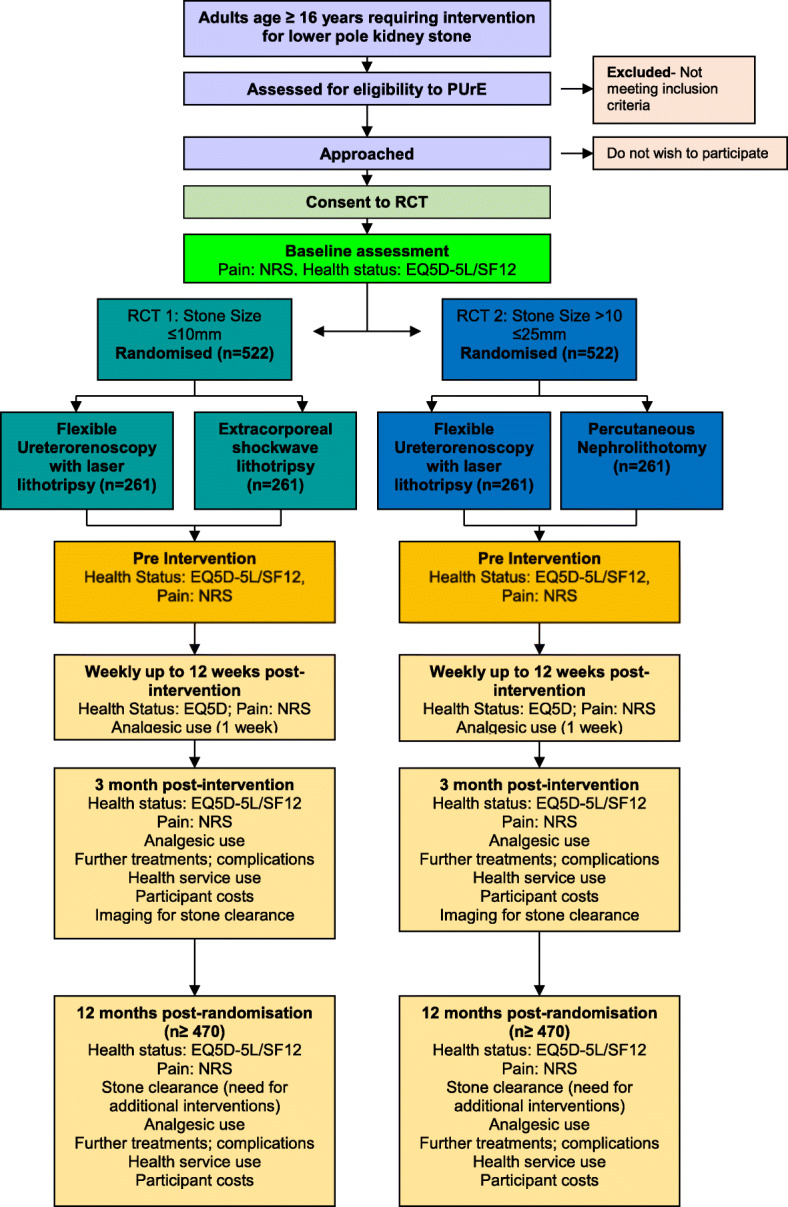
The clinical and cost effectiveness of interventions for stones in the lower pole of the kidney: the percutaneous nephrolithotomy (PCNL), flexible ureterorenoscopy (FURS) and extracorporeal shockwave lithotripsy (ESWL) for lower pole kidney stones randomised controlled trial (PUrE RCT). EQ-5D-5L EuroQol five dimensions five-level version questionnaire, NRS Numeric Rating Scale, SF-12 12-item short-form survey

The trial will comprise two separate (RCT1 and RCT2) pragmatic, multi-centre, patient-randomised, open-label superiority RCTs with an initial internal pilot phase. A summary of the trial design is shown in Fig. 3. RCT1 will compare FURS with ESWL, recruiting patients with stones of maximum dimension ≤10 mm. RCT2 will compare FURS with PCNL, recruiting patients with stones of maximum dimension >10 mm and ≤25 mm.

### Interventions to be evaluated

The experimental intervention FURS will be compared with the standard interventions ESWL and PCNL. All three interventions are currently in general use by urology departments throughout the UK NHS. This trial aims to test the interventions in a standard NHS setting so that the results are generalisable to current routine care in the UK. In line with this aim, all procedures will be delivered in NHS facilities and supervised by NHS staff trained and competent in the procedures. All participants will be under the care of a named consultant urologist who, as in standard NHS practice, will be responsible for planning and carrying out the allocated procedure and arranging follow-up. The surgical interventions FURS and PCNL will be carried out by a trained urologist, or by a trainee urologist under the supervision of a senior urologist. They will be supported by the standard team of ward and theatre staff and radiographers. In some centres, a specialist uro-radiologist will also assist with the procedures, particularly with access to the stone. The ESWL intervention can be delivered using any device approved for this purpose including both fixed-site and mobile lithotriptors. Delivery of the treatment will be according to local practice by staff trained in the procedure, typically a radiographer and nurse, and supervised by a urologist. The techniques and equipment used for FURS, PCNL and ESWL continue to evolve and hence will differ in detail between different surgeons and departments. The trial protocol will not mandate the use of any specific detailed technical method for each intervention, but as part of trial initiation of each site the standard procedure including equipment used for FURS, PCNL and ESWL for that site will be recorded on a trial proforma and updated with changes as necessary. It is anticipated that at 8–12 weeks post-intervention participants will receive imaging to assess stone clearance in accordance with usual standard of care.

For FURS, a thin (3-mm diameter) flexible endoscope (ureteroscope) is passed into the kidney via the natural urinary passages (urethra, bladder and ureter) and is used to directly see the stone. A laser fibre (typically 200 or 273 μm holmium laser fibre) is then passed through the working channel of the ureteroscope and laser energy used to fragment the stone within the kidney. Larger fragments can be retrieved with a wire basket device passed through the working channel whilst smaller fragments (<2 mm) may be left to pass spontaneously. Generally, the patient will pass remaining fragments in the urine during the week following the procedure. The procedure is performed as a day-case or with an overnight stay (2014 NHS average = 1.7 days) and usually requires general anaesthesia. A single dose of antibiotic to prevent infection is often given at the start of the procedure. The duration of the operation depends on size of stone but is typically 1.0–1.5 h. A temporary ureteral stent may be placed at the end of the procedure to protect against blockage of the ureter caused by swelling of its lining cells. The operating surgeon will monitor progress and degree of stone clearance during the procedure and this may be checked with a plain kidney x-ray afterwards. Possible harms of the procedure are urinary tract infection, bleeding and damage to the urinary system which may require a more prolonged period of stenting. The stent itself can cause pain and urinary symptoms such as increased urinary frequency and haematuria. For the purposes of the PUrE trial, FURS treatment is expected to be a single procedure in the great majority of cases. However, an additional procedure will be considered as part of the FURS treatment strategy in cases of technical complexity or larger stones as long as it takes place with 6 weeks of the initial FURS procedure. Any additional procedures will be recorded separately for trial purposes. Once stone clearance has been confirmed the ureteric stent will be removed as an outpatient procedure with local anaesthetic. Placement and removal of the stent will be considered part of the FURS treatment strategy in the PUrE trial.

ESWL involves the generation of an external acoustic (sound) pulse, called a shockwave, outside the body which is then focused onto the kidney stone through the patient’s flank skin, causing it to fragment. Stone fragments pass down the urinary tract spontaneously which may take a few weeks. It is routinely performed in an outpatient setting with analgesia, with or without sedation as required. A single dose of antibiotic may be given at the start of the procedure if there is thought to be a higher than normal risk of getting an infection afterwards. Each session lasts 1.0–1.5 h and stone fragmentation is monitored during the procedure and then by a plain x-ray (or other imaging as standard) taken at a follow-up appointment at approximately 3 weeks. For the PUrE trial, two separate ESWL treatments will be considered as part of the initial ESWL intervention strategy. These should take place within an 8-week period and each episode will be recorded separately for trial purposes. The treating urologist may, however, decide that further ESWL is not appropriate if stone fragmentation is insufficient following the first or second session. The first session of ESWL will be taken as the initial treatment point for the purposes of timing of outcome assessments. Possible harms of ESWL include urinary tract infection, visible bleeding in the urine and blockage of the ureter by the stone fragments. There is also a small risk of bruising surrounding the kidney.

PCNL is a surgical procedure to remove stones from the kidney by a direct approach. A small (10-mm) incision is made on the skin overlying the kidney through which a needle is passed into the urine collecting tube system of the kidney. This can be guided either by simultaneous ultrasound imaging of the kidney or by preliminary telescopic placement of a tube through the urethra, bladder and ureter. Contrast fluid can then be injected into the collecting system to guide the needle passage through the skin and into the kidney. Placement of the needle is planned using the available imaging (typically a computed tomography scan of the kidneys, ureters and bladder (CTKUB)) to give the best access to successfully remove the stone. For a stone in the lower pole of the kidney placement is usually into the lowermost part of the collecting system. Once the needle is satisfactorily placed, a flexible guide wire is then passed into the collecting system of the kidney and used to guide stretching (dilatation) of the needle track to make it wide enough for a hollow rigid access sheath to be passed creating a 10-mm wide channel between the skin and the urine collecting system of the kidney. A rigid metal telescope (nephroscope) can then be inserted down this channel into the kidney’s collecting system to see the stone and either retrieve it whole using graspers or to fragment the stone using a variety of energy delivery devices, most commonly an ultrasonic probe or pneumatic device. After the operation, the kidney is drained for a period by a tube placed either through the access channel or as a stent down the ureter into the bladder. In addition, a urinary catheter may be inserted to drain the bladder for a short period after the procedure. The operation is performed under general anaesthesia with a typical duration of 1–3 h depending on complexity, and patients usually stay in hospital for a few days (2014 NHS average stay = 4.6 days). Antibiotic treatment is frequently given at the start of the procedure and may be continued for a few days after if there is active infection. The drainage tubes are usually removed after 24–48 h without the need for further anaesthesia. Stone clearance is monitored during the procedure and, if necessary, by a plain x-ray (or other imaging) before discharge from hospital. Possible harms include urinary infection, bleeding (which may be severe), and inadvertent puncture of other organs. For the PUrE trial, a single PCNL treatment is expected to be required to completely remove stones up to 25 mm.

Apart from randomised allocation of the initial intervention and participant completion of questionnaires, the PUrE trial does not seek to change or impose any specific protocol regarding the clinical management of participants recruited at each trial site. The trial will however record relevant aspects of participant care during their involvement in the trial up to 12 months after randomisation and obtain patient-reported outcome measures. In particular, trial participants undergoing any of the stone treatments under test may require further interventions either to correct harms arising from the initial intervention or because of inadequate stone clearance by the initial intervention. The circumstances, nature and outcome of these additional procedures will be recorded, and patient -reported outcome measures obtained where possible before and after each additional intervention to assess the effect on health status.

### Trial population

The trial population will consist of adults (≥16 years old) presenting to NHS urology departments with a stone ≤25 mm in the lower pole of the kidney confirmed by non-contrast CTKUB. The patient and clinician must agree that active intervention is appropriate to remove the lower pole stone, and patients must be able to undergo either treatment for the specific stone size and be capable of giving informed consent which includes adherence with the requirement of the trial. Patients with multiple stones will be eligible provided all stones in the lower pole measure ≤25 mm in maximum dimension and, if there are stones of any size elsewhere in the urinary system, that the lower pole stone is the priority for treatment. A list of study sites is available on the study website (www.puretrial.org).

### Selection of participants

Clinicians will assess patients presenting with lower pole kidney stone. This will be aided by patient and clinician trial publicity material. A screening log documenting brief details of potentially eligible patients but without personal identifiers will be kept at each site to provide a summary of reasons for non-inclusion in the study to inform the Consolidated Standards of Reporting Trials diagram and assess generalisability of trial findings.

### Planned inclusion and exclusion criteria

#### Inclusion criteria

- Adults ≥16 years of age
- Lower pole stone ≤25 mm in maximum dimension with the decision to treat that stone
- Presence of stone previously confirmed by CTKUB
- Able and willing to undergo either treatment for the specified stone size
- Capacity to give informed consent to participate in the trial, which includes adherence to trial requirements

#### Exclusion criteria

- Pregnancy
- Patients with a co-existing stone that takes precedence in deciding treatment modality (such as an obstructing ureteric stone or large upper pole stone)
- Patients with health or other factors that are absolute contraindications to an intervention that they may be allocated
- Patients unable to understand or complete trial documentation

### Recruitment and trial procedures

#### Identifying participants

Patients with lower pole stones eligible for PUrE may be referred electively to urology departments having had a stone identified opportunistically by abdominal imaging or during investigation of urinary tract symptoms. Alternatively, they may present as an emergency with loin pain or infection. We will therefore inform clinical teams at each trial site of the target population backed up by trial publicity and trial summaries. At an appropriate point during their initial assessment, patients will be informed about the trial and given or sent information about the trial including a patient information leaflet with the contact details of the local research team. Patients will be given adequate time to consider participating in the study. They will have the opportunity to take study information away with them if desired, in which case the local research team will contact the patient after at least 24 h to determine their interest. If the patient is interested, the research team will confirm eligibility and discuss the individual’s possible participation with the clinical team. If both patient and clinical team agree regarding participation, arrangements will be made for consent to study, randomisation and clinical discussion and timing of allocated treatment. Wherever possible these processes will be arranged to take place together during one visit. Patients who decline, those who are ineligible or those for whom one of the possible allocated treatments is unsuitable will be recorded without identifiers on a screening log.

#### Informed consent

The patient information leaflet explains that the trial is investigating the effectiveness of active interventions for stones in the lower pole of the kidney. Patients will be informed that, depending on stone size, the trial will investigate whether the use of FURS will be superior to ESWL (stones ≤10 mm) or to PCNL (stones >10 mm and ≤25 mm). Signed informed consent forms will be obtained from the participants in all centres. Participants who cannot give informed consent (e.g. due to incapacity) will be not be eligible for participation. The participant’s permission will be sought to inform their general practitioner that they are taking part in this trial. We will also take optional consent for agreement to be approached for further studies on kidney stones, and for long-term follow-up through their local and central NHS clinical records after their active trial participation has finished.

#### Randomisation and allocation

Eligible and consenting participants, who have previously had a CTKUB to confirm the presence and size of stone, will be randomised dependent on the stone size using the telephone interactive voice response randomisation application or via the web-based application — both hosted by the fully registered UK Clinical Research Collaboration Clinical Trials Unit at the Centre for Healthcare Randomised Trials (CHaRT) Health Services Research Unit in Aberdeen, UK. Participants with a stone ≤10 mm will be entered into RCT1 and randomised to either FURS or ESWL. Participants with a stone >10 mm and ≤25 mm will be entered into RCT2 and randomised to either FURS or PCNL. Participants will then follow the standard care pathway for the allocated treatment. All the treatments allocated by randomisation in the study are used in routine clinical practice and in this pragmatic trial patients are expected to be treated using normal local clinical pathways and guidelines. The only trial-specific interventions apart from randomised treatment allocation will be participant completion of outcome questionnaires. Adherence to the allocated intervention will be monitored by the trial office team.

#### Blinding

There can be no blinding of participants, clinical staff or the central trial team to the allocated trial arm due to the differing nature of the interventions.

#### Follow-up procedures

Eligible patients who have given signed informed consent to participate in the study will be asked to complete the EQ-5D-5L, 12-item short-form survey (SF-12), pain score (by the Numeric Rating Scale (NRS)) and use of analgesic questions at baseline prior to randomisation. A baseline clinical case report form (CRF) will also be completed that will include stone size measured as the maximum dimension on a CTKUB. They will then be randomised to either one of the interventions dependent on the size of the lower pole stone and placed upon the appropriate waiting list. Waiting time duration for the trial interventions will be recorded and monitored. Participants will be asked to complete the pain score (NRS), EQ-5D-5L and use of analgesic questions at a number of fixed and variable time points during their trial participation. Fixed points will be baseline, just prior to initial intervention, weekly up to 12 weeks after initial intervention (FURS, PCNL and first ESWL session) and at 12 months post-randomisation. Variable points after 12 weeks will be just prior and 1 week after any additional intervention (including planned additional sessions of ESWL and removal of stent) and during any other hospital admissions related to treatment of their lower pole kidney stone (such as admissions for pain control or infection). At 12 weeks following the initial intervention, and at 12 months post-randomisation, participants will be asked to complete questions relating to their primary and secondary care use and their associated travel. At 12 months post-randomisation participants will be asked to additionally complete the SF-12. Participants will also be given the opportunity to complete an EQ-5D-5L at their discretion throughout the duration of the trial. A subset of 100 participants will receive the Cambridge Renal Stone Patient Reported Outcome Measure [34], a disease-specific health-related quality of life tool, at baseline, pre-intervention and at 12 weeks post-intervention.

Reminders may be used; for the earlier time points this may be a text message or email on the day that the questionnaire is due and, for the later time points (e.g. 12 weeks and 12 months), this reminder will be sent approximately 2 weeks and 4 weeks after the questionnaire is due.

We will offer and use all methods of delivery and collection of questionnaires and reminders including use of research teams for time points associated with hospitalisation, postal mail, email, web-based, telephone and SMS text, taking into account each participant’s stated preferred means of receiving and completing the measures. Participants will be sent a voucher (of modest value) as a token of appreciation for completion and return of the questionnaires.

CRFs collecting information on received care process and outcome will be completed by site research teams at baseline, following each initial and subsequent additional intervention (including planned additional ESWL sessions and stent removal), at 12 weeks post-intervention, after any additional stone-related treatments (e.g. admissions due to pain or infection) and 12 months post-randomisation. These will be entered at site into the web-based trial management platform.

To measure the secondary clinical outcome of stone clearance, participants will have kidney imaging at between 8 and 12 weeks according to clinical need and participant convenience. We will state preference for imaging by CTKUB during site initiation, but renal ultrasound and plain x-ray will be acceptable according to patient preference, safety and local practice. We will ask local site clinical teams (radiologist/urologist) to state whether there is: complete clearance of the target stone from the urinary tract defined as no further action or observation required for that stone; acceptable clearance where observation is required but no intervention planned; and unacceptable clearance where further intervention will be required. The maximum dimension of the largest remaining fragment in millimetres will also be recorded.

#### Withdrawal procedures

Participants are free to withdraw consent to participate at any time. Outcome data derived from medical records will be collected for those that withdraw unless the participant specifically withdraws their consent for this. All data collected up to the point of withdrawal will be retained and used in the analysis. Failure to undergo allocated treatment either because of participant preference or change of circumstance will not result in withdrawal and the participant will continue to participate in trial procedures unless consent to the trial is withdrawn.

### End of trial

The end of participation in the trial for each participant is defined as the final data capture to answer the research question: the 12-month post-randomisation questionnaire and CRF or the 12-week post-intervention questionnaire and CRF, whichever comes last.

### Safety

The PUrE trial involves procedures for treating lower pole stones that are all well established in current NHS clinical practice. Adverse effects may occur during or after any type of surgery. We will monitor serious adverse events and the local Principal Investigator or their delegate at the site will categorise these as expected or unexpected. Only serious unexpected adverse events related to the trial interventions or death of a participant will be notified to the Sponsor and Ethics Committee.

Hospital visits (planned or unplanned) associated with further interventions or complications of treatment (e.g. expected adverse events) due to the lower pole stone will be recorded as an outcome measure but will not be reported as serious adverse events. Other serious adverse events related to the intervention will not be reported a serious adverse event, but will be recorded in the CRFs. Planned primary care or hospital visits for conditions other than those associated with the lower pole stone will not be collected or reported. All deaths for any cause (related or otherwise) will be recorded on the serious adverse event form.

Within PUrE, ‘related’ is defined as an event that occurs as a result of a procedure required by the protocol, whether or not it is either the specific intervention allocated at randomisation or administered as an additional intervention as part of normal care.

### Provisions for post-trial care

Standard care is provided within the UK NHS.

### Outcome measures

#### Primary outcome measure

The primary outcome measures are health status measured by EQ-5D-5L AUC to 12 weeks post-intervention, based upon EQ-5D-5L completion at fixed time points (at baseline (recruitment), just prior to initial intervention (FURS, PCNL or first session of ESWL), weekly up to 12 weeks after initial intervention) and at variable time points (just prior to any additional intervention (including planned additional ESWL sessions and removal of stent) and once during hospitalisation for adverse events related to treatment (e.g. pain and infection)), and incremental cost per QALY gained at 12 months post-randomisation based on the estimated NHS costs and participant responses to the EQ-5D-5L (including an additional time point at 12 months).

#### Secondary outcome measures

Secondary outcome measures include the severity of pain as measured by the NRS (completed with EQ-5D-5L), generic health profile as measured by the SF-12 (completed at baseline and 12 months), use of analgesics (completed with NRS and EQ-5D). In addition, secondary outcome measures include stone clearance measured between 8 and 12 weeks following the initial intervention using renal imaging (CTKUB preferred, but plain x-ray and ultrasound are also acceptable), the maximum dimension of the largest fragment of the treated stone in millimetres, the need for additional treatment (carried out or planned) at 12 weeks post-initial treatment and 12 months post-randomisation, complications during the initial intervention, and intervention-related complications at 12 weeks (categorised by Clavien–Dindo classification) following treatment and up to 12 months post-randomisation. All these will be measured by site staff and entered on the CRF.

We will also record NHS primary and secondary care resources used and their costs, patient costs (out of pocket expenses), and time off work up to 12 months post-randomisation. Data gathered from completion of CRFs by site staff and participant questionnaire at 12 weeks following initial treatment and 12 months post-randomisation will also be recorded.

### Data collection and processing

#### Measuring outcomes

Outcome data will be collected throughout the trial for each participant from consent until 12 months following randomisation. See Table 1 for the schedule of events.

**Table 1:** Source and timing of measures

		Timing					
		Intervention (PCNL or first session ESWL/FURS)					
		Weeks post intervention				Additional intervention (pre and post if >12 weeks) or treatment-related hospitalisation	Post- randomisation 12 months
Outcome measure	Source	Baseline^a^	Pre	1 to 11	12		
Health status EQ-5D-5L	PQ	✓	✓	✓	✓	✓	✓
Pain	PQ	✓	✓	✓	✓	✓	✓
Health profile SF12	PQ	✓	✓		✓		✓
Use of analgesics	PQ	✓	✓	✓	✓	✓	✓
Stone clearance (imaging)	CRF				✓^b^		✓
Additional interventions received	CRF&PQ				✓	✓	✓
Complications	CRF&PQ				✓	✓	✓
NHS primary and secondary healthcare use	CRF, PQ		✓		✓	✓	✓
Participant costs	PQ		✓		✓		✓

*CRF* case report form, *PQ* participant completed questionnaire

^a^Baseline is after informed consent has been given but prior to randomisation

^b^stone imaging performed at 8-12 weeks post treatment

#### Data processing

Data collected locally will be input at sites by the local research team. Staff in the trial office will work closely with the local research teams to ensure data are as complete and accurate as possible. Participant questionnaires will be sent from and returned to the trial office in Aberdeen with the exception of the 1- and 2-week questionnaires, which may be distributed by the local research teams. Extensive range and consistency checks will further enhance the quality of the data.

#### Confidentiality

Data are stored in accordance with Good Clinical Practice and with the UK Data Protection Acts 1998 and 2018.

### Sample size, proposed recruitment rate and milestones

#### Sample size

The primary outcome is the AUC measured from multiple completions of the EQ-5D-5L by each participant up to 3 months following initial intervention (FURS, PCNL or first session of ESWL). In order to detect a standard deviation difference of 0.3, with 90% power, and alpha set at 5%, 235 participants per group (470 in total) are required. Such a difference in generic health status is considered clinically relevant and, in terms of treatment effect size, in the small to medium range as observed in other clinical studies. To allow for the anticipated 10% of participants for whom outcome data will be completely missing, and therefore the AUC cannot be calculated, it is proposed to randomise 522 participants in both RCT1 and RCT2, giving a total trial population of 1044 participants.

#### Recruitment rates

We plan to recruit the trial population from approximately 50 NHS centres across the UK, each recruiting an average of one participant per month to either RCT1 or RCT2. Our plan is to achieve the target of 1044 participants (522 to each RCT) over a 52-month recruitment window.

### Statistical analysis

The primary outcome — health status AUC — will be generated for each participant using the trapezoidal rule. Data for participants who have missed a scheduled time point will be estimated using a multiple imputation approach to make use of partial outcome data. Sensitivity analyses will be conducted to assess the robustness of the treatment effect estimate to these approaches. The primary outcome measure will be analysed using linear regression with adjustment for design variables. Secondary outcomes will be analysed using generalised linear models with adjustment for design and baseline variables as appropriate. Subgroup analyses will explore the possible modification of treatment effect by important factors (centre, participant body mass index, stone size (maximum dimension and volume), stone density on CTKUB (Hounsfield units), skin to stone distance). We will also explore within each allocated group whether technical factors modify the treatment effect (access sheath versus no access sheath and digital versus non-digital instrument (FURS); fixed-site versus mobile device (ESWL); calibre of access track (PCNL)). This will be done by including treatment-by-factor interactions in the model and they will be classified as exploratory analyses. All analyses will initially be performed on an intention-to-treat basis, although we will consider additional analysis groups such as per-protocol if indicated. The main statistical analyses will be based on all participants as randomised, irrespective of subsequent compliance with the treatment allocation. All treatment effects sizes will be summarised by estimates and 95% CIs from the appropriate models. From the feasibility phase we will report estimates of recruitment rates and potential participant availability, together with appropriate CIs. There are no planned interim outcome analyses; all analyses will occur following completion of trial follow-up. Interim analyses will be performed if requested by the Data Monitoring Committee (DMC).

All analyses will follow a carefully documented statistical analysis plan. RCT1 and RCT2 will be analysed entirely separately. The Trial Steering Committee (TSC) and the independent DMC will be asked to review and comment on the statistical analysis plan prior to analysis. We propose that progress and monitoring of the two RCTs will be undertaken within the same DMC and TSC. The team propose that each study will be analysed once completed. The DMC and TSC will meet before recruitment begins to agree its terms of reference and other procedures.

### Economic evaluation

An economic evaluation to assess the relative efficiency of trial care pathways will be an integral part of the study. A within -trial analysis [35, 36] as well as a simple Markov model [37, 38] to extrapolate the analysis beyond the RCT follow-up period will be considered. The perspective of the analysis will be that of the NHS and personal social services [39]. The analysis will rely on participant responses to the EQ-5D-5L to estimate quality-adjusted life years (QALYs) at 12 months. Resource use and costs will be estimated for each participant. The evaluation will consider the costs of the care pathways that patients receive. This will include costs of the interventions (ESWL, FURS, and PCNL) and the cost of simultaneous and consequent use of primary and secondary NHS services (including additional interventions received) by participants. Personal costs such as purchase of medications, particularly analgesics, will be estimated. As the clinical condition commonly affects people of working age, time off work will be also recorded to estimate indirect costs (e.g. human capital approach). The incorporation of indirect costs into the economic evaluation is debatable; however, the collection of these data will open the possibility to include these costs into the analysis or report them separately following reporting practice at the time of analysis.

Participant-level resource use data will be captured for the initial intervention and any subsequent admissions/treatments required through to 12 months post-randomisation using CRFs. Patient primary care services resource use as well as medications will be recorded using a patient questionnaire delivered at 12 weeks post-intervention and at 12 months post-randomisation. Special attention will be taken on questionnaire wording to minimise recall time overlapping (and hence avoid double counting). In addition, the patient questionnaires will collect data on time off work. Each resource use event will be valued using appropriate unit prices obtained from national sources, including NHS reference costs [40], and the unit cost of health and social care [41]. The British National Formulary [42] will be used to obtain unit costs to value medications, and published wage categories used to value time off work. Total NHS costs will be summed for each patient to 12 months post-randomisation.

#### Participant costs

Participant costs will comprise self-purchased health care (e.g. prescription and over the counter medication). Information will be collected using the 12-week post-intervention and 12-month post-randomisation questionnaires. Participants will be asked for information on travel costs incurred by visits to their general practitioner, hospital doctor or other health care provider.

#### NHS resource use

The use of secondary care services following the treatment period will be collected using participant questionnaires and CRFs. Information on outpatient visits (participant questionnaire at 12 weeks post-intervention and 12 months post-randomisation), readmissions and additional interventions relating to the use and consequences of the interventions being compared will be recorded on the CRF. The use of primary care services such as prescription medications, contacts with primary care practitioners (e.g. general practitioners and practice nurses) will be collected via the ‘health care utilisation questions’ administered at 12 weeks post-intervention and 12 months post-randomisation.

#### Cost effectiveness

Cost effectiveness will be measured in terms of costs of the treatment care pathways and QALYs at 12 months post-randomisation for the within-trial analysis. Mean NHS costs, patient costs and QALYs will be compared between randomised groups at 12 months. Incremental costs and QALYs will be estimated for FURS versus ESWL (for participants with stones ≤10 mm) and for FURS versus PCNL (for participants with stones >10 mm and ≤25 mm stones) using linear regression with adjustment for design variables and baseline values as appropriate. The final decision on what regression model to use is data-dependent. However, as the RCT is planned to involve 50 recruitment sites from the UK the use of multi-level regression models will be considered [43]. Uncertainty surrounding joint estimates of incremental cost and effects will be characterised and presented graphically using cost-effectiveness acceptability curves [44, 45]. Guidelines for economic evaluation advocate for a long enough time horizon to consider all cost and consequences relevant for the analysis [39]. In order to assess longer term cost effectiveness, a simple Markov model will be developed using available data on recurrence rates and extrapolation of costs and effects out to 5 years post-randomisation.

### Trial management and oversight arrangements

The trial office is in the CHaRT based within the Health Services Research Unit, University of Aberdeen, and provides day-to-day support for the local recruitment sites. The PUrE trial office team aims to meet formally at least monthly during the course of the trial to ensure smooth running and trouble-shooting.

#### Project Management Group

The trial is supervised by its Project Management Group consisting of the grant holders and representatives from the trial office. The Project Management Group aim to meet in person or by teleconference every 6 months on average.

#### Trial Steering Committee

The trial is overseen by a TSC. The TSC comprises four independent members, N. Parr (Chair), L. Harper, T. Everitt and J. Hussey. CHaRT has adopted the TSC charter adapted from the DAMOCLES charter for DMCs and suggests to the independent TSC members that they adopt the terms of reference contained within. The TSC will meet approximately yearly.

#### Data Monitoring Committee

An independent DMC will be convened. The DMC is comprised of three independent members, S. Payne (chair), D. Douglas and R. Hills. The trial statistician contributes as appropriate and the Chief Investigator and/or a nominated delegate may contribute to the open session of the meetings as appropriate. CHaRT has adopted the DAMOCLES charter for DMCs and suggests to the independent DMC members that they adopt the terms of reference contained within.

The Committee meets regularly to monitor the unblinded trial data and serious adverse events and to make recommendations as to any modifications that are required to be made to the protocol or the termination of all or part of the trial.

### Dissemination

All RCTs conducted by CHaRT have a commitment to publish the findings of the research. At a minimum, this trial will have a results paper published in a peer-reviewed medical or scientific journal. If all grant holders and research staff fulfil authorship rules, group authorship may be appropriate for some publications under the collective title of ‘the PUrE Trial Group’. If one or more individuals have made a significant contribution above and beyond other group members, but where all group members fulfil authorship rules, authorship may be attributed to the named individual(s) and the PUrE Trial Group.

To safeguard the integrity of the main trial, reports of explanatory or satellite studies will not be submitted for publication without prior arrangement from the Project Management Group.

We intend to maintain interest in the trial by publication of PUrE newsletters at intervals for staff and collaborators. Once the main report has been published, a lay summary of the findings will be sent in a final PUrE newsletter to all involved in the trial, including trial participants.

## Discussion

The PUrE trial is a large, multi-centre, pragmatic RCT to determine the clinical and cost effectiveness of surgical interventions for stones in the lower pole of the kidney. The primary outcome for the study is participant-reported health status (AUC up to 12 weeks post-intervention calculated from multiple measurements of the EQ-5D-5L).

It is not possible to blind the participants or clinicians in PUrE due to the differing nature of the interventions (invasive versus non-invasive) and their requirements (e.g. anaesthesia, multiple treatments). This may be a source of bias in the trial; however, this is limited by the pragmatic nature of the study, randomisation and concealment of allocation.

The main practical challenge of the study has been participant recruitment. Stone disease has a high recurrence rate [1], and many of those approached will have had a lived experience of treatment and this may influence their willingness to be randomised. Screening data indicates that, of those approached, 30% of patients in RCT1 have a preference for treatment that prevented them from entering the trial, and this figure is higher in RCT2 (41%). A qualitative study was instigated to investigate this further to try and identify some best practises from key sites to disseminate to all researchers. However, we have experienced some difficulties implementing the study, largely due to staff feeling uncomfortable about recruitment consultations being audio-recorded.

Due to the unexpected issues with recruitment, trial timelines have been extended by 18 months to allow us to reach the target for RCT1. Early on in the study it became apparent that RCT2 would not recruit to target as the patient population is not as large for RCT2 (stones >10 and ≤25 mm) as it is for RCT1. This is reflected in the screening data; there have been considerably fewer patients screened for RCT2 compared to RCT1 (252 versus 1036 at the time of writing).

Other than the difficulties previously discussed there have been no major issues conducting the PUrE study.

### Trial status

The first participant was recruited in May 2016 and the trial is currently open to recruitment in 50 UK centres. The current protocol version is version 02, 2 September 2019. Recruitment is due to complete on 30 September 2020.

## Data Availability

Data may be available for collaborators on request to the Chief Investigator, SM (pure@abdn.ac.uk).

## Abbreviations

AUC: Area under the curve
CHaRT: Centre for Healthcare Randomised Trials
CI: Confidence interval
CRF: Case report form
CTKUB: Computed tomography scan of the kidneys, ureters and bladder
DMC: Data Monitoring Committee
EQ-5D-5L: EuroQol five dimensions five-level version questionnaire
ESWL: Extracorporeal shockwave lithotripsy
FURS: Flexible ureterorenoscopy
NHS: National Health Service
NRS: Numeric Rating Scale
PCNL: Percutaneous nephrolithotomy
QALY: Quality-adjusted life year
RCT: Randomised controlled trial
RR: Relative risk
SF-12: 12-item short-form survey
TSC: Trial Steering Committee
